# Association between dietary intake of creatine and female reproductive health: Evidence from NHANES 2017–2020

**DOI:** 10.1002/fsn3.4135

**Published:** 2024-04-30

**Authors:** Sergej M. Ostojic, Tonje Holte Stea, Stacey J. Ellery, Abbie E. Smith‐Ryan

**Affiliations:** ^1^ Applied Bioenergetics Lab, Faculty of Sport and PE University of Novi Sad Novi Sad Serbia; ^2^ Department of Nutrition and Public Health University of Agder Kristiansand Norway; ^3^ Faculty of Health Sciences University of Pecs Pecs Hungary; ^4^ Department of Health and Nursing Sciences University of Agder Kristiansand Norway; ^5^ The Ritchie Centre Hudson Institute of Medical Research Clayton Victoria Australia; ^6^ Department of Obstetrics and Gynaecology Monash University Clayton Victoria Australia; ^7^ Department of Exercise and Sport Science University of North Carolina Chapel Hill North Carolina USA

**Keywords:** creatine, dietary intake, fetal macrosomia, NHANES, oligomenorrhea

## Abstract

The hormonal changes in women influence creatine dynamics, emphasizing its potential importance during menstruation, pregnancy, postpartum, menopause, and postmenopause. Yet, limited research explores creatine's impact on female reproductive health at the population level. Our study investigated the relationship between dietary creatine intake and reproductive health indices in US women using data from the 2017–2020 National Health and Nutrition Examination Survey (NHANES). We extracted a dataset containing females aged 12 years and above who provided details about their reproductive health and dietary habits. Daily creatine intake was quantified as a relative amount (mg per kg body mass) and did not include creatine from dietary supplements and pharmacological agents. A daily requirement for dietary creatine for healthy women was employed to classify respondents into two separate subpopulations: (1) suboptimal intake of creatine (<13 mg per kg body mass per day) or (2) recommended intake (dietary creatine ≥ 13 mg per kg body mass per day). A total of 4522 female participants from the NHANES study (age 44.5 ± 20.5 years) provided data on their reproductive health and dietary intake. The average daily creatine intake for the group was 10.5 ± 10.8 mg per kg body mass. The odds ratio for having irregular periods in women consuming ≥13 mg of creatine per kg body mass daily (recommended intake) compared to those with suboptimal intake was 0.75 (95% CI, from 0.66 to 0.86), indicating a significant association between higher intake of dietary creatine and lower risk of oligomenorrhea (*p* < .001). Moreover, women consuming less than 13 mg of creatine per kg body mass faced an increased risk of fetal macrosomia (OR 1.26; *p* = .04), pelvic infection (OR 1.68; *p* = .01), hysterectomy (OR 1.42; *p* < .001), oophorectomy (OR 1.54; *p* < .001), and receiving hormone replacement therapy (OR 1.26; *p* = .02). Consuming a creatine‐rich diet has been linked to lower risks of reproductive issues in US women aged 12 and above. Those consuming ≥13 mg of creatine per kg body mass daily showed notably lower risks of irregular menstrual periods, obstetric conditions, and pelvic pathology. Further studies are needed to confirm these potential benefits.

## Graphical abstract

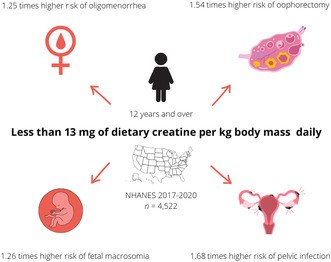

## 1 INTRODUCTION

Creatine is considered a semi‐essential nutrient critical for maintaining normal energy homeostasis across the life course, from early development into adult life (Ostojic & Forbes, 2022; Ostojic, Grasaas, & Cvejic, 2023; Ostojic, Ratgeber, et al., 2023). As energy‐demanding cells, both ovum and spermatozoa are highly dependent on creatine during maturation and fertilization, with creatine supporting normal pregnancy and newborn health in both animal models and human studies (for a detailed review, see Muccini et al., 2021; Ostojic, Forbes, et al., 2022; Ostojic, Stea, et al., 2022). Several preliminary human studies have demonstrated the importance of creatine acquisition for optimal reproductive health, including sperm viability and capacitation (Umehara et al., 2018), better pregnancy outcomes (Dickinson et al., 2016), and postnatal brain development (Korovljev et al., 2023). Given the hormonal alterations affecting creatine dynamics in women, the consumption of creatine through diet might hold particular significance during menstruation, pregnancy, postpartum, menopause, and postmenopausal phases (Smith‐Ryan et al., 2021). However, there is still a paucity of studies examining the links between creatine intake and female reproductive health at the population level. In this study, we examined the associations between dietary creatine intake and indices of reproductive health in US women aged 12 years and older using data from the 2017–2020 National Health and Nutrition Examination Survey (NHANES).

## 2 METHODS

The NHANES population comprises noninstitutionalized individuals living in all 50 US states, as well as Washington DC (CDC/National Center for Health Statistics, 2023a). Due to the COVID‐19 pandemic, data for the NHANES 2017–2020 round were compiled by combining information collected from 2019 to March 2020 with data from the NHANES 2017–2018 cycle to create a nationally representative sample of 15,560 respondents, with 50.4% being women. For this study, we extracted a dataset that included female participants aged 12 years and older, who provided information on their reproductive health and dietary intake. The age of 12 years was taken as the average age of menarche for the NHANES program; no data on female reproductive health were available for younger women in NHANES databases. The information related to reproductive health was obtained from the NHANES 2017–2020 Questionnaire Data for Reproductive Health (data published in August 2021). The questions on reproductive health included menstrual history, pregnancy history, hormone replacement therapy use, and other related reproductive conditions (CDC/National Center for Health Statistics, 2023b). These questions were asked at the Mobile Examination Center (MEC), by trained interviewers, using the Computer‐Assisted Personal Interview system as part of the MEC interview. Both proxy interviewers and interpreters were permitted for these questions. Information regarding dietary intake was derived from the NHANES 2017–2020 Dietary Data interview files (data published in July 2022). Individual data files containing detailed information about each food and beverage item consumed were used to calculate creatine intake from meat‐ and milk‐based food sources (US Department of Agriculture food codes from 11100000 to 28522000), as described previously (Ostojic, Grasaas, & Cvejic, 2023; Ostojic, Ratgeber, et al., 2023). Daily creatine intake was quantified as a relative amount (mg per kg body mass) and did not include creatine from supplements and pharmacological agents. The approval to conduct NHANES 2017–2020 was granted by the US National Center for Health Statistics Ethics Review Board. The NHANES complex sampling design was utilized for data management. Data series were initially assessed for normality using the Kolmogorov–Smirnov test and visual inspection. Independent *t*‐tests and chi‐squared tests were employed to compare mean values and proportions across creatine intake categories (below or above the recommended intake), respectively. A daily requirement for dietary creatine for healthy women (13 mg [5.0 mmol] per kg body mass per day for women in the 20‐ to 39‐year‐old age group; Brosnan & Brosnan, 2007) was employed to classify respondents into two separate subpopulations: (1) suboptimal intake of creatine (<13 mg per kg body mass per day) or (2) recommended intake (dietary creatine ≥ 13 mg per kg body mass per day). Odds ratios (OR) were calculated to quantify the strength of the association between dietary creatine intake and reproductive health in subpopulations with different creatine intakes. Binomial logistic regression analyses were also used to evaluate the association between primary exposure (daily creatine intake) and outcome variables (reproductive health indicators, see below). The crude regression models were further adjusted for a defined set of co‐variates; Model 1 (dietary factors) included vitamin A (μg), folic acid (μg), vitamin D (μg), calcium (mg), zinc (mg), and iron (mg), and Model 2 (demographics) included generation, race, education level, income, and body mass index. Data were analyzed using IBM SPSS Statistics for Mac (Version 24.0), with the significance level set at *p* ≤ .05.

## 3 RESULTS

A total of 4522 female NHANES respondents provided information about reproductive health and dietary intake data. Demographic and basic dietary characteristics of the study sample are shown in Table 1. Among the respondents, regular periods in the past 12 months were reported by 2260 respondents (50.0%), while 2055 (45.4%) disclosed irregular periods (oligomenorrhea); the responses for 207 participants were missing or unavailable. A total of 3053 women (67.5%) reported pregnancy at least once in their lifetime, with 59 women (1.3%) being at the time of data collection. The mean number of pregnancies, including current pregnancy, live births, miscarriages, stillbirths, tubal pregnancies, or abortions, was 3.45 (95% confidence interval [CI], from 3.35 to 3.54); the number of deliveries resulting in live birth was 2.59 children (95% CI, from 2.52 to 2.65). In addition, 277 respondents (6.1%) attempted to become pregnant over a period of at least a year without becoming pregnant. During pregnancy, 274 women (6.1%) were told by a health professional to have gestational diabetes. A total of 533 women (11.8%) delivered a baby that weighed 4082 grams or more at birth, a threshold indicating fetal macrosmia (Akanmode & Mahdy, 2023). Moreover, 163 women (3.6%) reported having ever been treated for pelvic inflammatory disease and 620 women (13.7%) reported having ever used female hormones (excluding birth control methods or use for infertility). Finally, 813 women (18.0%) reported having had hysterectomy, and 423 (9.4%) women reported having both ovaries were removed (either when the uterus was removed or at another time).

**TABLE 1:** Demographic and basic dietary characteristics of the study sample (*n* = 4522).

Variable	
Age, mean ± SD (years)	44.5 ± 20.5
Generation (%)	
Preteens, 12.0–12.9 years	2.1
Teenagers, 13.0–17.9 years	11.1
Adults, 18.0–64.9 years	67.1
Elderly, ≥65.0 years	19.7
Race (%)	
Mexican American	12.4
Other Hispanic	10.0
Non‐Hispanic White	34.5
Non‐Hispanic Black	27.3
Other race, including multiracial	15.9
Ratio of family income to poverty, mean ± SD	2.5 ± 1.6
Income less than the poverty level (%)	21.3%
Body mass index, mean ± SD (kg/m^2^)	30.0 ± 8.5
Obesity (%)	43.0
Energy intake, mean ± SD (kcal/day)	1847 ± 783
Protein, mean ± SD (g/day)	67.8 ± 32.6
Carbohydrate, mean ± SD (g/day)	218.2 ± 10.2
Total sugars, mean ± SD (g/day)	95.8 ± 61.4
Dietary fiber, mean ± SD (g/day)	14.6 ± 9.2
Total fat, mean ± SD (g/day)	76.7 ± 40.2
Total saturated fatty acids, mean ± SD (g/day)	24.5 ± 14.3
Cholesterol, mean ± SD (mg/day)	267.4 ± 213.3

**FIGURE 1:**
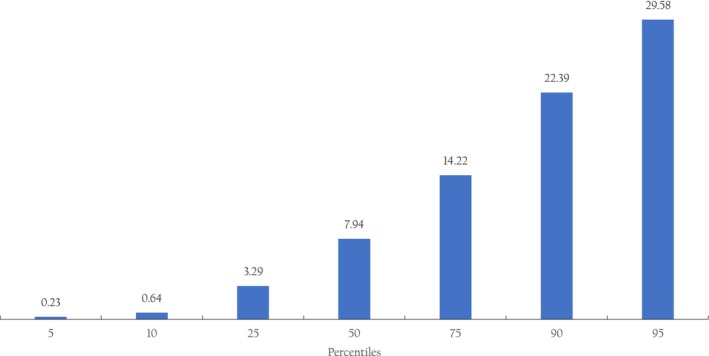
Percentiles of daily creatine intake (mg per kg body mass) across the sample (*n* = 4522). The daily recommended intake of dietary creatine for healthy women is 13 mg per kg body mass per day (Brosnan & Brosnan, 2007).

The average daily intake of creatine across the sample was 10.5 ± 10.8 mg per kg body mass (95% CI, from 10.2 to 10.8). The percentiles of daily creatine intake are depicted in Figure 1. The respondents with suboptimal intake of dietary creatine (<13 mg per kg body mass per day) significantly outnumbered respondents with recommended intake (71.1% vs. 28.9%; *p* = .004). Daily creatine intake was significantly different across generations (*p* < .001), with the mean creatine intake highest in preteens (13.2 ± 12.1 mg per kg body mass), followed by teenagers (11.2 ± 10.8 mg per kg body mass), and adult women (10.6 ± 11.4 mg per kg body mass), with the lowest amounts consumed in women aged 65 years and over (9.2 ± 8.3 mg of creatine per kg body mass). The highest daily intake of creatine (164.71 mg per kg body mass) was found in a 20‐year‐old nonobese woman who consumed creatine exclusively from meat‐based sources. A total of 712 respondents (15.7%) and 1027 respondents (22.7%) consumed no creatine from meat and milk sources, respectively.

Women with regular periods during the past 12 months consumed significantly more creatine per day than those with irregular periods (11.1 ± 11.9 mg/kg body mass vs. 9.8 ± 9.5 mg/kg body mass; *p* < .001). The odds ratio for having irregular periods in women consuming ≥13 mg of creatine per kg body mass daily (recommended intake) compared with those with suboptimal intake was 0.75 (95% CI, from 0.66 to 0.86), indicating a significant association between higher intake of dietary creatine and lower risk of oligomenorrhea (*p* < .001). In addition, women consuming less than 13 mg of creatine per kg body mass daily had a higher risk of fetal macrosomia (OR 1.26, 95% CI, from 1.01 to 1.57; *p* = .04), pelvic infection (OR 1.68, 95% CI, from 1.13 to 2.48; *p* = .01), hysterectomy (OR 1.42, 95% CI, from 1.18 to 1.71; *p* < .001), oophorectomy (OR 1.54, 95% CI, from 1.29 to 1.97; *p* < .001), and receiving hormone replacement therapy (OR 1.26, 95% CI, from 1.03 to 1.55; *p* = .02) compared with their higher‐intake peers, respectively. No differences in odds of having gestational diabetes and failed pregnancy were shown between the two subsamples of creatine consumers.

A crude binomial logistic regression revealed a significant association between higher creatine intake and lower risk of oligomenorrhea (*p* < .001), with each additional milligram of creatine per kg body mass consumed daily resulting in a 1.2% reduction in oligomenorrhea rates. The association remained significant even after adjusting the regression model for other dietary factors relevant to reproductive health (Model 1) and demographic variables (Model 2) (*p* < .05). Logistic regression results for other indices of reproductive health are depicted in Table 2.

**TABLE 2:** Logistic regression (*B* coefficients) for the relationship between dietary creatine intake and reproductive health indices in the crude model, Model 1 (adjusted for dietary intake of vitamin A, folic acid, vitamin D, calcium, zinc, and iron), and Model 2 (adjusted for generation, race, education level, income, and body mass index).

Variable	Crude model	Model 1	Model 2
Oligomenorrhea	−.012*	−.012*	−.008*
Failed pregnancy	−.006	−.010	−.002
Pelvic infection	−.021*	−.020*	−.015
Gestational diabetes	.003	.001	.007
Fetal macrosomia	−.004	−.005	−.001
Hysterectomy	−.015*	−.015*	−.012*
Oophorectomy	−.022*	−.021*	−.019*
Hormone replacement therapy	−.013*	−.012*	−.011

* Statistical significance at *p* < .05.

## 4 DISCUSSION

To our knowledge, this is the first population‐based study that has evaluated the links between dietary creatine intake and female reproductive health. Our study revealed that the daily creatine consumption was below the recommended level in a nationally representative sample of US women aged 12 years and older recruited during the NHANES 2017–2020 round. Women in our sample consumed 10.5 mg of creatine per kg body mass per day on average, with seven out of 10 women (71.1%) consuming creatine below recommended amounts, with intakes declining with age. Interestingly, women who consumed food‐derived creatine ≥ 13 mg per kg body mass per day (recommended intake) had a significantly lower risk of irregular menstrual periods, obstetric conditions, and pelvic pathology, suggesting that more creatine taken from food relates to better female reproductive health.

Female reproductive health is highly dependent on the successful regulation of cellular bioenergetics, utilizing various fuel sources to support the divergent needs of oocytes and neighboring tissues (Seli et al., 2014). As a main component of high‐energy phosphate pathways, creatine is considered to be crucial for female reproductive health during all life stages. For instance, the ovary and other reproductive organs (including the placenta) have a high expression of creatine‐specific enzymes/transporters and total creatine content, highlighting the role of this compound for reproductive health (Muccini et al., 2021; Philip et al., 2023; Wyss & Kaddurah‐Daouk, 2000). Creatine levels are maintained by endogenous synthesis and an omnivorous diet (Ostojic & Forbes, 2022). Providing dietary creatine has been shown to be beneficial for female reproductive health in various experimental models, with maternal dietary creatine intake improving egg production and quality in laying quails (Al‐Shammari et al., 2023), protecting the fetal brain from hypoxic–ischemic encephalopathy in rodents (for a detailed review, see Tran et al., 2021), and reducing the incidence of overlays of low birth weight piglets (Vallet et al., 2013). Only few human studies have evaluated the effects of dietary creatine intake on reproductive systems, where creatine recovered normal fetal development in a pregnant woman with creatine deficiency syndrome (Alessandrì et al., 2020), and improved pelvic functional status in women with stress‐predominant urinary incontinence (Takacs et al., 2023). Additionally, menstrual disturbances seem to be more prevalent among women who do not include creatine‐containing foods in their diets (Griffith & Omar, 2003). Although the present results are preliminary, the findings from prior studies advocate for a more comprehensive investigation into the potential links between creatine consumption and female reproductive health.

Our study contributes to the existing literature by implementing a population‐wide approach and effectively controlling for a few potential confounding variables. It showcases a robust association between higher dietary creatine intake and better reproductive health in women aged 12 years and older. We have demonstrated a significantly lower risk of several female reproductive disorders, including oligomenorrhea, fetal macrosomia, pelvic infection, hysterectomy, and oophorectomy, in women consuming ≥13 mg of creatine per kg body mass daily as compared to their low‐intake counterparts. Furthermore, the link between dietary creatine and reproductive health remained strong even after adjusting for demographics and dietary factors, suggesting a protective effect of consuming more creatine in this domain. Consuming an adequate amount of dietary creatine perhaps upholds cellular energy circuits in critical organs during specific stages of a female's reproductive life, including pregnancy and menopause (Ellery et al., 2016). Moreover, dietary creatine could aid in preserving cell hydration during the luteal phase of menstruation (Moore et al., 2023), which has been associated with positive effects on reducing menstrual discomfort (Torkan et al., 2021). By acting as an antioxidant, creatine could also alleviate oxidative stress and modulate ovarian and oviductal function to enhance reproductive performance (Al‐Shammari et al., 2023). Recognizing that female reproductive disorders pose a significant public health and economic challenge (Ezeh et al., 2016), promoting a diet rich in creatine could be recommended as an affordable and straightforward public health strategy to address the impact of these conditions. Still, a majority of women in our NHANES sample (71.1%) consumed creatine below recommended amounts, which is in line with previous studies describing highly prevalent creatine malnutrition across different populations (Ostojic, 2021; Ostojic, Forbes, et al., 2022; Ostojic, Stea, et al., 2022). This could lead to the consideration of forward‐thinking public strategies aimed at ensuring an adequate supply of creatine in food systems, incorporating low‐dose supplementation and food fortification.

Although this study presents promising findings, it is crucial to consider several limitations. This involves using self‐reported data to gather information on reproductive health and dietary intake, which might introduce bias and impact result accuracy. A somewhat arbitrary margin for recommended creatine intake was chosen due to the current lack of sufficient data to establish concrete recommendations. The required amount of creatine for women might be influenced by factors such as the presence of dietary precursors, age, pregnancy and lactation, the body's ability to naturally produce creatine, and genetic disorders affecting creatine requirements. Specifically, it would be interesting to analyze the relationships between peri‐ and postmenopausal women with lower female reproductive demands. For this study, we decided not to include creatine intake from supplements, as they are infrequently used among females, as indicated in prior research (Knapik et al., 2016). A further limitation lies in the cross‐sectional design of this study, which might not entirely reflect the regular dietary patterns due to variations in eating habits over time. Moreover, the absence of supplementary biomarkers for dietary creatine exposure (including creatine levels in the target tissues and/or circulation) might restrict the accurate assessment of creatine intake. We did not analyze the potential influence of other factors that might affect the risk of reproductive disorders, including hormone imbalances, environmental factors (e.g., exposure to endocrine‐disrupting compounds), and other chronic diseases. Therefore, our findings should not be construed as establishing a cause‐and‐effect relationship between creatine intake and reproductive health. It is strongly recommended to use a longitudinal study design to minimize the likelihood of reverse causation bias.

## 5 CONCLUSION

Consuming a diet rich in creatine has shown an association with a lower risk of reproductive disorders in women aged 12 and above in the United States. Women who consumed greater than or equal to 13 mg of food‐derived creatine per kg body mass per day exhibited a notably reduced risk of irregular menstrual periods, obstetric conditions, and pelvic pathology. Additional epidemiological and interventional studies are needed to confirm the potential advantages of creatine‐rich foods and/or dietary supplements in promoting female reproductive health.

## AUTHOR CONTRIBUTIONS

**Sergej M. Ostojic:** Conceptualization (equal); data curation (equal); formal analysis (equal); investigation (equal); methodology (equal); project administration (equal); software (equal); writing – original draft (equal). **Tonje Holte Stea:** Investigation (equal); methodology (equal); project administration (equal); supervision (equal); validation (equal); writing – review and editing (equal). **Stacey J. Ellery:** Investigation (equal); methodology (equal); project administration (equal); supervision (equal); validation (equal); writing – review and editing (equal). **Abbie E. Smith‐Ryan:** Investigation (equal); methodology (equal); project administration (equal); supervision (equal); validation (equal); writing – review and editing (equal).

## ACKNOWLEDGEMENTS

None.

## CONFLICT OF INTEREST STATEMENT

SMO, SJE, and ASR serve as members of the Scientific Advisory Board on creatine in health and medicine (AlzChem LLC). SMO co‐owns patent “Supplements Based on Liquid Creatine” at the European Patent Office (WO2019150323 A1). SMO has received research support related to creatine during the past 36 months from the Ministry of Education, Science, and Technological Development; Provincial Secretariat for Higher Education and Scientific Research; Alzchem Group AG; ThermoLife International; and Hueston Hennigan LLP. SMO does not own stocks and shares in any organization. ASR serves as a Scientific Advisor for Create Wellness, a creatine‐based gummy. THS declares no known competing financial interests or personal relationships that could have appeared to influence the authorship of this paper.

## ETHICS STATEMENT

Study approval statement: The ethics approval was granted by the US National Center for Health Statistics Ethics Review Board (Continuation of Protocol #2011–17, effective through October 26, 2017, and Protocol #201801, effective beginning October 26, 2017). Consent to participate statement: Written informed consent was obtained from all respondents to participate in the study. The research was conducted ethically following the World Medical Association Declaration of Helsinki.

## Data Availability

Data will be made available on request.
